# The impact of autoimmune comorbidities on multiple sclerosis progression: insights from a longitudinal single-center study

**DOI:** 10.1007/s00415-025-13351-2

**Published:** 2025-09-03

**Authors:** Derya Aslan, Sabrina Bourabia, Bernd Kowall, Agne Straukiene, Konstantin Fritz Jendretzky, Franz Felix Konen, Thomas Skripuletz, Aksel Siva, Mehmet Fatih Yetkin, Tim Hagenacker, Christoph Kleinschnitz, Refik Pul, Jelena Skuljec

**Affiliations:** 1 Department of Neurology, University Medicine Essen, Center for Translational Neuro- and Behavioral Sciences (C-TNBS), Hufelandstr. 55, 45147 Essen, Germany; 2 Medical Faculty, Institute for Medical Informatics, Biometry and Epidemiology, University Duisburg-Essen, Essen, Germany; 3 Department of Neurology, Torbay and South Devon NHS Foundation Trust, University of Plymouth, Torquay, Plymouth, UK; 4 Hannover Medical School, Department of Neurology, Hannover, Germany; 5 Cerrahpaşa School of Medicine, Department of Neurology, Clinical Neuroimmunology Unit & MS Clinic, Istanbul University, Istanbul, Turkey; 6 Faculty of Medicine, Department of Neurology, Erciyes University, Kayseri, Turkey

**Keywords:** Multiple sclerosis, Autoimmune diseases, Comorbidities, Disease activity, Disease-modifying therapy

## Abstract

Multiple sclerosis (MS) is a chronic autoimmune disease that affects the central nervous system. The presence of other conditions alongside MS imposes a substantial personal and socioeconomic burden. In particular, the impact of comorbid autoimmune diseases (AID) on MS outcomes remains uncertain. In this retrospective longitudinal study, we examined the prevalence of AID in a cohort of patients with different types of MS. For patients with relapsing–remitting MS (RRMS), negative binomial and linear regression models were used to assess adjusted associations between the presence of AID and various other clinical parameters of MS disease. A total of 861 MS patients were included, of whom 148 (17.2%) had one or more comorbid autoimmune diseases (AID). The majority of the patients with MS and AID were women (79.1%). The most prevalent AID was autoimmune thyroiditis (58.1%), followed by psoriasis (16.2%) and type 1 diabetes mellitus (11.5%). Among the 662 RRMS patients, we observed minimal changes in key metrics such as disease-modifying therapies (DMT), relapse rates, steroids dosages, plasma exchanges, or changes in the expanded disability status scale (EDSS) for those with AID. For example, the incident rate of relapses in DMT-naïve patients with AID was nearly identical to that of those without AID (incident rate ratio = 0.92, 95% confidence interval: 0.81 – 1.04). Our findings highlight the notable co-occurrence of AID among patients with MS and suggest that these comorbidities do not affect the progression of RRMS, regardless of DMT use. Reassessing this in large, multicenter, prospective, long-term studies is essential.

**Supplementary Information:**

The online version contains supplementary material available at 10.1007/s00415-025-13351-2.

## Introduction

Multiple sclerosis (MS) is a chronic immune-mediated disease that affects the central nervous system (CNS), leading to demyelination and axonal degeneration. It is the most common cause of non-traumatic neurological disability in young adults aged 18–40 years, affecting about 2.8 million people worldwide [1]. The exact cause is still unknown, but it is believed to involve multiple factors, including genetics, the environment, and potential infectious agents [1, 2].

At present, there is no cure for MS. Treatment strategies aim to slow long-term disability progression by reducing inflammatory attacks and preventing future relapses. Studies consistently support the benefits of early initiation of treatment [3, 4]. Nonetheless, the diverse clinical manifestations and treatment responses, the wide range of disease-modifying therapies (DMT), and the absence of precise diagnostic criteria and specific biological markers for diagnosis and prognosis pose challenges for healthcare providers [5]. It is crucial to thoroughly consider all drug- and patient-related factors to choose the most appropriate personalized therapeutic approach [5].

The presence of comorbidities, referring to the burden of chronic illnesses other than the primary disease [6], is a critical patient-related aspect associated with delays in MS diagnosis, accelerated physical and cognitive impairments, reduced quality of life, and higher mortality rates [6–8]. Therefore, early screening and management of comorbidities should be integrated into MS care [6, 9]. Among all comorbid conditions in MS, autoimmune diseases (AID) have been identified as the top priority to be addressed, given their significant impact on affected individuals, the healthcare providers and system, and overall society [6, 10]. Several studies have explored the association between MS and concurrent systemic or organ-specific AID. Some reported a higher rate of particular AID among people with MS and their first-degree relatives [11–18], while others could not identify this link [18–20]. It remains unclear whether people with MS have an increased susceptibility to develop AID [17, 21].

The co-occurence of AID and MS may indicate a shared underlying pathogenesis, possibly offering a unique target for therapy. Additionally, certain DMT for MS may contribute to the development of secondary AID [22, 23]. The underrepresentation of MS patients with AID in clinical trials highlights the need for more information on the efficacy and safety of interventions for this specific population [6, 24]. Our recent review of existing literature on the positive and negative effects of approved and currently studied DMT for MS in the context of various coexisting AID emphasizes the necessity for further research in this area [25], particularly in understanding the impact of AID as prognostic factors for MS outcomes and whether the presence of comorbid AID might influence DMT decision-making processes [6, 10].

In this cohort study, we examined the prevalence and types of comorbid AID in patients with various forms of MS. We hypothesized that individuals with comorbid AID experience higher relapse rates, utilize a greater total number of DMT, show a more substantial increase in disability, and are more frequently treated with steroids or undergo plasma exchange. To investigate this, we conducted a comprehensive analysis of the correlation between AID and clinical parameters in patients with RRMS, considering both the entire patient population and those who remain untreated.

## Material and methods

### Study population and procedures

We conducted a retrospective evaluation of 862 adult patients diagnosed with MS according to the 2017 revised McDonald criteria for clinically isolated syndrome (CIS), relapsing–remitting (RRMS), and primary progressive MS (PPMS). The 2023 revised diagnostic criteria for radiologically isolated syndrome (RIS) were applied for RIS patients. The diagnosis of secondary progressive MS (SPMS) followed the guidelines of the German Society of Neurology, requiring confirmation of MS diagnosis according to the 2017 revised McDonald criteria, including a relapse-independent progression over 12 months (https://dnvp9c1uo2095.cloudfront.net/cms-content/030050_living_Guideline_MS_V7.1_240105_1704444034393.pdf). Tumefactive MS (TMS) was diagnosed following analysis of brain biopsies. Patients received treatment at our tertiary care center at the University Medicine Essen in Germany between December 2017 and September 2021. The therapy administration was carried out independently of enrollment and in accordance with the latest summary of product characteristics. Demographic and clinical details including sex, age, body mass index, smoking status, disease duration, expanded disability status scale (EDSS), number of relapses, number of DMT used, use of steroids, number of plasmapheresis cycles, and presence and type of comorbid AID were recorded during the follow-up periods for each patient.

The diagnosis of an AID was confirmed by analyzing archived reports and laboratory data. We consistently inquired about the co-occurrence of comorbidities, especially AID, during the subjects’ first visit and at all follow-up appointments. Patients undergoing alemtuzumab therapy were closely monitored due to the increased risk of developing secondary autoimmune disorders [22]. Those who developed secondary autoimmunity were excluded from the study. The disease duration, EDSS, and number of relapses were thoroughly documented at each clinical visit and evaluated from the onset of MS until the end of the observation period (follow-up) period. The “first EDSS” refers to the initial value determined, while the “last EDSS” represents the final recorded value at the most recent follow-up before the data were analyzed. The incidence rate of relapses was calculated as the total number of relapses divided by the observation period.

The DMT encompassed a range of medications including: interferon-β−1a i.m. and s.c., interferon-β−1b, glatiramer acetate, teriflunomide, dimethyl fumarate, fingolimod, cladribine, ocrelizumab, ofatumumab, rituximab, daclizumab, natalizumab, and alemtuzumab.

All procedures adhered to relevant laws and institutional guidelines and received approval from the ethics committee (22–10,594-BO). We used the STROBE reporting guideline [26] to draft this manuscript and the STROBE reporting checklist [27] when editing.

### Statistical analyses

The statistical analyses were performed using SAS 9.4 software (SAS Institute, Cary, USA). To determine the incidence rate (IR), we calculated the number of new events observed during the follow-up period and divided this by the sum of the times at risk of the event for all subjects. We utilized negative binomial regression models to analyze outcome variables that have non-negative integer values. This approach enabled us to calculate incidence rate ratios (IRR) along with the corresponding 95% confidence intervals (CIs), allowing to identify associations between autoimmune status and key outcomes in MS (the frequency of relapses and the use of DMT) and comparison between two groups: those diagnosed with AID and those without. An IRR of 1 indicates that the incidence rates are identical in both groups, suggesting no significant association between AID and the MS outcomes. In our analysis, we selected negative binomial models over Poisson models to address issues of data overdispersion. The analysis included two models: a basic crude model and a more refined model that adjusts for key variables such as age at onset, sex, and time elapsed from the first manifestation of MS to the end of the follow-up period.

Linear regression models were fitted to assess the total annual steroid dose, number of plasma exchanges, and delta EDSS as outcomes. For each outcome, 1000 bootstrap samples of size 621 were drawn from the original data set with replacement. The beta coefficient for the association between autoimmune status and each outcome was estimated for each bootstrap sample. The mean, 2.5 percentile, and 97.5 percentile of the distribution of the 1,000 beta coefficients were computed to provide the point estimate with a 95% CI. The linear regression models were adjusted for age at onset, sex, and time from the first manifestation of MS to the end of follow-up in months. Additionally, sensitivity analyses were conducted, excluding cases with autoimmune thyroiditis.

### Artwork and illustrations

**Fig. 1:**
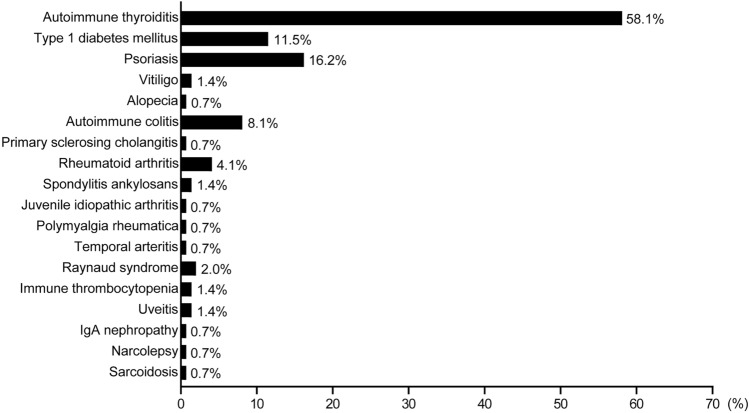
The proportion of autoimmune comorbidities in patients with MS. The distribution and types of comorbid autoimmune disease (AID) are presented for all MS patients who have at least one comorbid AID (*n* = 148). This cohort includes all disease courses: radiologically isolated syndrome, clinically isolated syndrome, relapsing–remitting multiple sclerosis, secondary progressive multiple sclerosis, and primary progressive multiple sclerosis

**Fig. 2:**
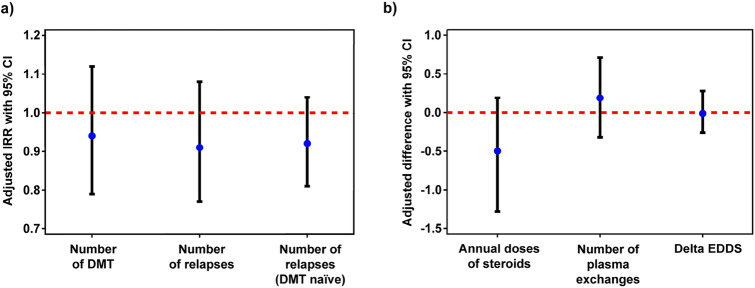
The association between autoimmune comorbidities and clinical parameters in RRMS. **a** Negative binomial regression models were utilized to predict the usage of disease-modifying therapies (DMT), the number of relapses, and the number of relapses within 12 months after the RRMS onset in untreated patients who have comorbid autoimmune disease (AID). The results are reported as incidence rate ratios (IRR) with 95% confidence intervals (CI). **b** The outcomes from 1000 bootstrapped linear regression models illustrate the correlation between AID and clinical outcomes in patients with RRMS. Delta EDSS: difference between the last and the first recorded EDSS. Both models (a and b) have been adjusted for the age at the onset of RRMS, sex, and the duration from the initial MS manifestation to the end of the follow-up period

The graph for Fig. 1 was created using GraphPad Prism 9 (Version 9.5.1, GraphPad Software, San Diego, California, USA), while Fig. 2 was generated using SAS 9.4. The final artworks were designed using Adobe Illustrator (Adobe Inc., San Jose, California, USA).

## Results

### Total cohort

The data in Table 1 provide a comprehensive overview of our cohort, comprising 861 individuals affected by RIS, CIS, and diverse types of MS, including RRMS, SPMS, and PPMS. Notably, the majority of our study participants were women (68.3%, *n* = 587). RRMS was the most prevalent type, representing 72.3% of the cohort, and these patients experienced the highest number of total relapses (4.4 ± standard deviation (SD) 3.5). The SPMS group had the longest follow-up period (68.3 ± SD 154.7 months) and disease duration (291.7 ± SD 122.8 months), as well as the highest mean age at the last neurological visit (59.1 ± SD 8 years) and EDSS scores at disease onset and study end (5.3 ± SD 1.8 and 6.0 ± SD 1.6, respectively). The PPMS group exhibited the oldest age at disease onset (44.1 ± SD 12 years).

**Table 1:** Characteristics of the entire cohort—summary of patient demographics and clinical parameters *RIS* radiologically isolated syndrome, *CIS* clinically isolated syndrome, *RRMS* relapsing–remitting multiple sclerosis, *SPMS* secondary progressive multiple sclerosis, *PPMS* primary progressive multiple sclerosis, *TMS* tumefactive multiple sclerosis, *SD* standard deviation, *EDSS* expanded disability status scale, *ARR* annual relapse rate, *AID* autoimmune disease, *N/A* not applicable

Diagnosis	RIS	CIS	RRMS	SPMS	PPMS	TMS	Total
% (N)	0.7 (6)	6.6 (57)	72.3 (622)	11.6 (100)	8.4 (72)	0.5 (4)	100 (861)
Female sex, % (N)	50.0 (3)	71.9 (41)	69.9 (435)	70.0 (70)	50.0 (36)	50.0 (2)	68.3 (587)
Age at the last visit Mean ± SD (median), years	49.2 ± 14.5 (49.5)	39.1 ± 10.2 (39.0)	42.8 ± 12.1 (42.0)	59.1 ± 8.0 (59.0)	57.2 ± 10.0 (57.0)	40.2 ± 14.3 (41.0)	45.6 ± 13.1 (46.0)
Age at syndrome/MS onset Mean ± SD (median), years	N/A	34.7 ± 10.6 (35.0)	30.2 ± 10.9 (29.0)	33.3 ± 11.6 (30.0)	44.1 ± 12.0 (44.0)	37.0 ± 14.4 (36.5)	32.1 ± 11.7 (30.0)
Syndrome/MS duration Mean ± SD, months	N/A	27.4 ± 27.1 (16.0)	139.2 ± 128.9 (103.0)	291.7 ± 122.8 (289.0)	153.6 ± 184.2 (107.0)	29.5 ± 14.8 (29.0)	149.9 ± 142.0 (109.5)
First EDSS Mean ± SD (median)	N/A	1.7 ± 1.6 (1.5)	2.2 ± 1.6 (2.0)	5.3 ± 1.8 (5.7)	4.6 ± 1.7 (4.0)	2.0 ± 2.0 (0.0)	2.7 ± 2.0 (2.0)
Last EDSS Mean ± SD (median)	N/A	1.5 ± 1.4 (1.5)	2.5 ± 1.8 (2.0)	6.0 ± 1.6 (6.0)	5.3 ± 1.6 (5.5)	1.3 ± 0.6 (1.0)	3.1 ± 2.2 (2.5)
ARR Mean ± SD (median)	N/A	1.2 ± 1.9 (0.7)	0.7 ± 1.2 (0.4)	0.2 ± 0.26 (0.2)	NA	1.0 ± 1.2 (0.4)	0.6 ± 1.2 (0.4)
Number of relapses Mean ± SD (median)	N/A	1.3 ± 0.9 (1.0)	4.4 ± 3.5 (3.0)	4.3 ± 3.2 (4.0)	NA	2.0 ± 1.0 (1.0)	3.8 ± 3.5 (3.0)
AID % (N)	16.7 (1)	17.5 (10)	16.2 (101)	25.0 (25)	15.3 (11)	0 (0)	17.2 (148)
AID female % (N)	100 (1)	80.0 (8)	80.2 (81)	88.0 (22)	45.5 (5)	0 (0)	79.1 (117)
Age at AID diagnosis Mean ± SD (median), years	27.0 (27.0)	27.0 ± 6.9 (27.5)	26.8 ± 13.6 (26.0)	42.3 ± 15.8 (45.0)	32.7 ± 19.0 (35.0)	N/A	29.8 ± 15.1 (29.0)
Follow-up Mean ± SD (median), months	N/A	11.6 ± 12.2 (9.0)	47.2 ± 69.6 (34.0)	68.3 ± 154.7 (36.5)	30.8 ± 30.8 (25.0)	22.3 ± 20.4 (27.0)	46.0 ± 80.9 (31.0)

Comorbid AID were present across all MS phenotypes, with the exception of TMS. In the entire cohort, 17.2% of MS patients (*n* = 148) had at least one comorbid AID and the vast majority (79.1%) were female. AID were most prevalent in patients with SPMS, affecting 25% of individuals. This group had the highest percentage of affected females (88%) and developed AID at an average age of 42.3 ± 15.8 years. Patients with PPMS comprised the only clinical MS subtype where both male and female sexes were equally represented. In this subgroup, male patients accounted the majority (54.5%) of those impacted by AID (Table 1).

The distribution of comorbid AID by functional organ/system is detailed in Table 2 and Fig. 1. Among all MS patients, 88.6% had one AID, while 11.5% presented with two AIDs. The most prevalent AID was autoimmune thyroiditis (58.1%), followed by psoriasis (16.2%). Type 1 diabetes mellitus (11.5%) and autoimmune colitis (8.1%) were also frequently observed.

**Table 2:** Distribution and types of comorbid AID in the entire MS cohort including all disease courses The “AID cohort” comprises MS patients with at least one comorbid AID

AID	N	% in the AID cohort	% in the total MS cohort
**Presence of comorbid AID**			
One comorbid AID	131	88.6	15.2
Two comorbid AID	17	11.5	2.0
**AID classified by organ/system**			
Endocrine			
Autoimmune thyroiditis	86	58.1	10.0
Type 1 diabetes mellitus	17	11.5	2.0
Integumentary			
Psoriasis	24	16.2	2.8
Vitiligo	2	1.4	0.2
Alopecia	1	0.7	0.1
Gastrointestinal			
Autoimmune colitis	12	8.1	1.4
Primary sclerosing cholangitis	1	0.7	0.1
Rheumatological			
Rheumatoid arthritis	6	4.1	0.7
Spondylitis ankylosans	2	1.4	0.2
J uvenile idiopathic arthritis	1	0.7	0.1
Polymyalgia rheumatica	1	0.7	0.1
Cardiovascular			
Temporal arteritis	1	0.7	0.1
Raynaud syndrome	3	2.0	0.3
Hematologic			
Immune thrombocytopenia	2	1.4	0.2
Visual			
Uveitis	2	1.4	0.2
Urinary			
IgA nephropathy	1	0.7	0.1
Neurological			
Narcolepsy	1	0.7	0.1
Pulmonary			
Sarcoidosis	1	0.7	0.1

### RRMS cohort

In a subgroup of 622 patients with RRMS, 101 individuals (16.2%) also had comorbid AID. The proportion of females was higher in this group (80.2%), compared to RRMS patients without AID (67.9%; Table 3). Additionally, those with AID experienced MS symptoms at a slightly more advanced age, with a difference of around 1 year (31.1 ± 12.4 versus 30.1 ± 10.6 years). The presence of AID was associated with higher initial and final EDSS scores. However, the change in EDSS scores over time (delta EDSS) was only marginally higher in patients with AID (0.4 ± 1.2) than in those without AID (0.3 ± 1.3). This may be additionally connected with the longer MS disease duration period in the patients with AID, indicating no significant differences in disability progression between the groups (Table 3). Notably, RRMS patients with coexisting AID had a lower prevalence of smoking (32.0% versus 45.2%) and tended to have a higher BMI (27.9 ± 7.6 versus 25.7 ± 5.4) than those without AID (Table 3).

**Table 3:** Demographics and clinical parameters of RRMS patients classified based on the presence of comorbid autoimmune disease SD: standard deviation; EDSS: expanded disability status scale; PY: pack-years; BMI: body mass index; MS disease duration: period from the first manifestation of MS to the last neurological visit. Missing data:^a^ 2 values,^b^6 values,^c^ 22 values,^d^ 5 values,^e^ 16 values,^f^ 1 value,^g^ 19 values,^h^ 9 values,^i^ 47 values

Autoimmune disease		
	yes	no
% (*N*)	16.2 (101)	83.8 (521)
Female sex, % (*N*)	80.2 (81)	67.9 (354)
Age at the last visit, mean ± SD, years	45.7 ± 12.1	42.2 ± 12.0
Age at MS onset, mean ± SD (years)	31.1 ± 12.4	30.1 ± 10.6 ^a^
First EDSS, mean ± SD	2.6 ± 1.6 ^b^	2.1 ± 1.6 ^c^
Last EDSS, mean ± SD	3.0 ± 1.8 ^d^	2.4 ± 1.8 ^e^
Delta EDSS, mean ± SD	0.4 ± 1.2 ^b^	0.3 ± 1.3 ^c^
Disease (MS) duration, mean ± SD (months)	157.8 ± 113.3 ^f^	135.6 ± 131.4 ^f^
Smokers, % (N)	32.0 (32) ^f^	45.2 (227) ^g^
Smoking status (PY), mean ± SD	17.1 ± 12.7 ^f^	18.3 ± 14.9 g
BMI, mean ± SD	27.9 ± 7.6 h	25.7 ± 5.4 ^i^

The results indicated that even after adjusting for key variables such as age at the onset of MS, sex, and the duration between MS onset and follow-up, there were minimal differences in the number of DMT used (IRR = 0.94, 95% CI 0.79–1.12) or relapse rates (IRR = 0.91, 95% CI 0.77–1.08) between RRMS patients with AID and those without AID (Table 4 and Fig. 2a). Moreover, it is noteworthy that also in untreated RRMS patients, the relapse rate closely aligned with those of the entire RRMS cohort, regardless of AID status (IRR = 0.92, 95% CI 0.81–1.04; Table 4 and Fig. 2a).

**Table 4:** The results of negative binomial regression models used to predict key outcomes: the use of disease-modifying therapies (DMT), the number of relapses, and the number of relapses within 12 months after the MS onset in untreated patients with RRMS who also have comorbid AID The results are presented as incidence rate ratios (IRR) with 95% confidence intervals (CI).^a^ The model was adjusted for age at MS onset, sex, and the duration between the initial MS manifestation and the end of the follow-up period

		*N*	Number of DMT	Follow-up Mean ± SD, months	IRR_crude_ (95% CI)	IRR_adjusted_^a^ (95% CI)
**Autoimmune disease**	yes	100	2.65 ± 1.94	157.8 ± 113.3	0.96 (0.80–1.15)	0.94 (0.79–1.12)
	no	520	2.25 ± 1.58	135.6 ± 131.4	1	1

		*N*	Number of relapses	Follow-up Mean ± SD, months	IRR_crude_ (95% CI)	IRR_adjusted_^a^ (95% CI)
**Autoimmune disease**	yes	100	5.12 ± 4.07	157.8 ± 113.3	0.94 (0.79–1.12)	0.91 (0.77–1.08)
	no	520	4.29 ± 3.34	135.6 ± 131.4	1	1

		*N*	Number of relapses (DMT naïve)	Observation time, months	IRR_crude_ (95% CI)	IRR_adjusted_^a^ (95% CI)
**Autoimmune disease**	yes	61	1.20 ± 0.48	12	0.94 (0.83–1.06)	0.92 (0.81–1.04)
	no	241	1.28 ± 0.71	12	1	1

In our analysis, we observed that patients with RRMS and AID received a lower number of annual doses of steroids but a higher number of plasma exchanges compared to RRMS patients without AID. The average bootstrap estimate for the differences was − 0.50 (95% CI: − 1.28–0.19) for steroids and 0.19 (95% PI: − 0.32, 0.71) for plasma exchanges (Table 5). Additionally, we found no significant difference in the change in EDSS score (delta EDSS) between patients with AID and those without AID, with an average bootstrap estimate of 0.01 (95% CI: − 0.28–0.26; Table 5 and Fig. 2b).

**Table 5:** The outcomes of 1000 bootstrapped linear regression models used to explore the correlation between AID and various outcomes in patients with RRMS Delta EDSS difference between the last and the first recorded EDSS. ^a^ The model was adjusted for age at MS onset, sex, and the duration between the first manifestation of MS and end of follow-up (in months). ^b^ The 2.5% and 97.5% percentiles were used for the analysis

		N	Annual doses of steroids	Average bootstrap estimate with percentile interval ^a,b^
**Autoimmune disease**	Yes	100	1.85 ± 2.44	− 0.50 (− 1.28, 0.19)
	No	520	2.66 ± 6.56	
		**N**	**Number of plasma exchanges**	**Average bootstrap estimate with percentile interval **^**a,b**^
**Autoimmune disease**	Yes	100	0.87 ± 2.53	0.19 (− 0.32, 0.71)
	No	520	0.69 ± 2.11	
		**N**	**Delta EDSS**	**Average bootstrap estimate with percentile interval **^**a,b**^
**Autoimmune disease**	Yes	94	0.38 ± 1.21	− 0.01 (− 0.26, 0.28)
	No	498	0.29 ± 1.35	

We further categorized the RRMS patients according to their genetic backgrounds. Out of 622 individuals, we were able to classify 489 patients with known genetic backgrounds into three main groups based on their country of origin: Europeans, Asians, and Africans. Notably, 78% of the individuals identified as having European descent, with the majority (67.3%) being of German heritage. The Asian cohort comprised 13.1% (*n* = 64), while the African group represented only 2.2% (*n* = 11; Online Resource 4). However, further subgroup analyses based on different genetic backgrounds were not reliable due to small sample sizes, which resulted in wide confidence intervals.

### RRMS cohort, excluding patients with autoimmune thyroiditis

Based on previous research, a connection has been suggested between the deregulated anti-inflammatory mechanisms in MS and chronic autoimmune thyroid diseases [28]. This possible link may account for the high prevalence of chronic autoimmune thyroid diseases in our RRMS cohort, with 57 out of 101 individuals affected. Accordingly, we excluded patients with this condition and conducted analyses to explore the associations between other comorbid AID and factors such as the number of relapses, use of DMT, annual steroid dosage, plasma exchanges, and changes in EDSS score. A detailed overview of the patient demographics and clinical parameters is provided in Online Resource 1. The findings revealed only minor differences in these parameters between the groups, as outlined in Online Resource 2 and 3.

## Discussion

This study is the first to examine the association between MS and AID within a German MS patient cohort, providing new insights into the impact of AID as a risk factor for MS progression, particularly in patients who have not yet undergone treatment. Our findings indicate that 17.2% of all MS patients, including those with RIS, CIS, and all clinically definite MS phenotypes, also had AID, with 15.2% of patients presenting with one AID and 2% with two comorbid AID. This prevalence significantly surpasses the 4–9% range reported in large population-based studies [29–32]. Our data are in line with previous research on the prevalence of AID in MS patients [33, 34], and also demonstrate variations across specific MS phenotypes [34]. The high co-occurrence of MS and AID may be attributed to shared genetic, environmental, or behavioral risk factors [35], increased monitoring following an MS diagnosis [20], or the direct causation of one condition and/or its treatment on the other [6].

Recent research has revealed notable disparities in the incidence, prevalence, and progression of different MS subtypes between men and women [36, 37]. Our analysis, in alignment with previous studies, consistently demonstrates a higher prevalence of relapsing–remitting and secondary progressive MS forms in women, while the primary progressive course affects both sexes equally. Furthermore, a higher proportion of women are affected by comorbid AID across all MS disease phenotypes except for PPMS, reflecting the higher prevalence of AID in females observed in the general population as well [29, 30, 38]. These sex-based differences are likely influenced by complex interactions among genetic, hormonal, and environmental factors [36]. Further research into sex-specific disparities is crucial to enhance the efficacy of personalized treatment.

The average age at which AID are diagnosed in our cohort of MS patients was 29.8 years, considerably earlier than the general population’s average of 54 years as reported in a recent British study [30]. The earlier onset of AID symptoms in individuals with pre-existing autoimmune conditions may be attributed to heightened immune responses caused by greater genetic susceptibility and ongoing chronic inflammation. Multiple genetic risk variants for various AID, including MS, have been identified, impacting immune function [35] and potentially leading to accelerated immune aging, malfunction of immune effector cells, and irreversible tissue damage [39].

In our MS cohort, the most common coexisting AID was autoimmune thyroiditis, followed by psoriasis and type I diabetes mellitus. These results align with findings from other studies involving MS patients [15, 33, 40, 41], but they are considerably higher than those recently reported for the general German population [29]. The dysregulation of immune tolerance mechanisms, specifically the BACH2/PDCD5-FOXP3 pathways and Tregs, may be shared underlying factors between MS and Hashimoto’s thyroiditis [28].

To enhance statistical power, our further analysis focused on RRMS patients, the largest subgroup in our cohort. We found that comorbid AID were associated with slightly older age at MS onset and at most recent follow-up. Additionally, patients with AID had higher EDSS scores at both time points, but the change in EDSS scores over time did not differ significantly between the two groups. These results suggest that contrary to our hypothesis, the presence of comorbid AID is not associated with an earlier MS onset or faster MS progression.

Both smoking and obesity are established risk factors for both MS and AID [42–45]. Moreover, there is a report suggesting that smokers have a higher risk of developing AID after the onset of MS [46]. In our study cohort, we observed lower tobacco consumption among RRMS patients with AID compared to those without AID. The lack of a positive correlation may be due to an increase in other risk factors, such as the higher prevalence of obesity in this group, or to reasons that are currently unknown.

The existing literature on the impact of comorbid AID on MS outcomes is limited [6]. A previous report indicated that as the total number of various physical and mental comorbidities increases, the use of DMT decreases [47]. Additionally, some comorbid conditions have been associated with a higher risk of relapses [48, 49]. We have found no significant correlation between the presence of AID and either the number of DMT used or the relapse rate, within both the entire RRMS group and in untreated RRMS patients. Using a linear regression model with bootstrap estimation, we identified a negative correlation of comorbid AID with the annual usage of steroids and a positive correlation with the number of plasma exchange interventions. Furthermore, our predictive model has suggested similar increase in EDSS scores during follow-up for both patient groups, regardless of the presence of AID. It is important to note that we collectively analyzed all AID present in the RRMS cohort. However, a recent study from Sweden indicated an elevated risk of reaching EDSS scores of 6.0 and 3.0 in individuals with MS and type 1 diabetes mellitus or ulcerative colitis, respectively [50]. Taken together, our findings suggest that the presence of AID does not substantially influence the progression and disability worsening of RRMS.

It is important to point out that while patients with AID may exhibit similar clinical symptoms of MS as those without AID, this does not necessarily rule out the possibility of subtle pathological progression due to AID. Studies using magnetic resonance imaging have shown an association of certain AID, such as psoriasis, type 2 diabetes mellitus, thyroid diseases [51], and type 1 diabetes mellitus [52], with more severe brain injury and atrophy in MS patients, regardless of their clinical features. Therefore, large long-term studies should determine whether the presence of AID contributes to an elevated risk for clinical progression of MS over time.

Our study has certain limitations. The sample size was insufficient for detailed differentiation based on specific comorbid AID, the timing of AID onset/diagnosis in relation to MS onset/diagnosis, and each DMT category. Furthermore, this article did not consider the impact of comorbidity-related therapies, reasons for switching DMT, or the outcomes of comorbidities due to MS.

The study’s real-world approach adds strength to these findings, particularly the inclusion of untreated patients, which eliminates the confounding effects of MS therapies on autoimmune comorbidities. However, further multicenter, prospective studies with larger sample sizes are needed to better understand the specific interactions between individual AID and MS progression, as this could optimize treatment outcomes and improve the quality of life for patients.

The lack of a clear association between AID and faster disease progression should not downplay the clinical relevance of comorbidities in MS care. Comorbid AID present a substantial burden that requires vigilant screening and integration into holistic MS management strategies. This is especially true given the potential for subtle pathological progression that could manifest in long-term outcomes, such as brain atrophy or cognitive decline.

## Supplementary Information

Below is the link to the electronic supplementary material.

Supplementary file1 (PDF 293 KB)

Supplementary file2 (PDF 297 KB)

Supplementary file3 (PDF 124 KB)

Supplementary file4 (DOCX 19 KB)

## Data Availability

Anonymized study data will be shared upon reasonable request from qualified investigators.
