# Supplementary material for: The impact of autoimmune comorbidities on multiple sclerosis progression: insights from a longitudinal single-center study
Source: J Neurol. 2025 Sep 3;272(9):607. doi: 10.1007/s00415-025-13351-2 (PMC12408772; doi:10.1007/s00415-025-13351-2)
Supplement: Supplementary file 1 — Supplementary file1 (PDF 293 KB) [file 415_2025_13351_MOESM1_ESM.pdf]

Journal: Journal of Neurology (Springer Nature)

The Impact of Autoimmune Comorbidities on Multiple Sclerosis Progression: Insights from a Longitudinal Single-Centre Study

Derya Aslan[a], Sabrina Bourabia[a], Bernd Kowall[b], Agne Straukiene[c], Konstantin Fritz Jendretzky[d], Franz Felix Konen[d], Thomas Skripuletz[d], Aksel Siva[e], Mehmet Fatih Yetkin[f], Tim Hagenacker[a], Christoph Kleinschnitz[a], Refik Pul[a,*], Jelena Skuljec[a]

[a] University Medicine Essen, Department of Neurology, Center for Translational Neuro- and Behavioral Sciences (C-TNBS), Essen, Germany.

[b] Medical Faculty, University Duisburg-Essen, Institute for Medical Informatics, Biometry and Epidemiology, Essen, Germany.

[c] Torbay and South Devon NHS Foundation Trust, Department of Neurology, Torquay, United Kingdom; University of Plymouth, Plymouth, United Kingdom.

[d] Hannover Medical School, Department of Neurology, Hannover, Germany.

[e] Istanbul University, Cerrahpaşa School of Medicine, Department of Neurology, Clinical Neuroimmunology Unit & MS Clinic, Istanbul, Turkey.

[f] Erciyes University, Faculty of Medicine, Department of Neurology, Kayseri, Turkey.

*Corresponding author: Prof. Refik Pul, MD; E-mail: refik.pul@uk-essen.de

	**Autoimmune disease**	
	yes	no
**% (N)**	44	521
**Age, mean ± SD, years**	44.7 ± 12.0	42.2 ± 12.0
**Age at MS onset, mean ± SD (years)**	31.7 ± 13.5 [a]	30.1 ± 10.6 [b]
**Male sex, % (N)**	12 (27.3%)	167 (32.1%)
**Follow-up, mean ± SD (months)**	138.0 ± 102.2 [a]	135.6 ± 131.4 [a]
**First EDSS, mean ± SD**	2.50 ± 1.53 [c]	2.11 ± 1.56 [d]
**Last EDSS, mean ± SD**	2.74 ± 1.59 [e]	2.40 ± 1.75 [f]

Online Resource 1. Characteristics of RRMS patients, classified based on the presence of comorbid autoimmune disease. Patients with autoimmune thyroiditis were excluded from the analyses. SD: standard deviation; EDSS: expanded disability status scale; Follow-up: duration from the first manifestation of MS to the last neurological visit. Missing data: [a] 1 value, [b] 2 values, [c] 6 values, [d] 22 values, [e] 5 values, [f] 16 values.
