# Supplementary material for: The impact of autoimmune comorbidities on multiple sclerosis progression: insights from a longitudinal single-center study
Source: J Neurol. 2025 Sep 3;272(9):607. doi: 10.1007/s00415-025-13351-2 (PMC12408772; doi:10.1007/s00415-025-13351-2)
Supplement: Supplementary file 2 — Supplementary file2 (PDF 297 KB) [file 415_2025_13351_MOESM2_ESM.pdf]

Journal: Journal of Neurology (Springer Nature)

The Impact of Autoimmune Comorbidities on Multiple Sclerosis Progression: Insights from a Longitudinal Single-Centre Study

Derya Aslan[a], Sabrina Bourabia[a], Bernd Kowall[b], Agne Straukiene[c], Konstantin Fritz Jendretzky[d], Franz Felix Konen[d], Thomas Skripuletz[d], Aksel Siva[e], Mehmet Fatih Yetkin[f], Tim Hagenacker[a], Christoph Kleinschnitz[a], Refik Pul[a,*], Jelena Skuljec[a]

[a] University Medicine Essen, Department of Neurology, Center for Translational Neuro- and Behavioral Sciences (C-TNBS), Essen, Germany.

[b] Medical Faculty, University Duisburg-Essen, Institute for Medical Informatics, Biometry and Epidemiology, Essen, Germany.

[c] Torbay and South Devon NHS Foundation Trust, Department of Neurology, Torquay, United Kingdom; University of Plymouth, Plymouth, United Kingdom.

[d] Hannover Medical School, Department of Neurology, Hannover, Germany.

[e] Istanbul University, Cerrahpaşa School of Medicine, Department of Neurology, Clinical Neuroimmunology Unit & MS Clinic, Istanbul, Turkey.

[f] Erciyes University, Faculty of Medicine, Department of Neurology, Kayseri, Turkey.

*Corresponding author: Prof. Refik Pul, MD; E-mail: refik.pul@uk-essen.de

			N	Number of relapses	Incidence rates (per month)	IRR_{crude} (95% CI)	$IRR_{adjusted}$ [a] (95% CI)
**Autoimmune disease**	yes		44	4.91 ± 3.84	0.0356	1.01 (0.79 – 1.30)	0.99 (0.78 – 1.26)
	no		519	4.29 ± 3.34	0.0316	1	1
			**n**	**Number of DMT**	**Incidence rates (per month)**	**IRR_{crude} (95% CI)**	**$IRR_{adjusted}$ [a] (95% CI)**
**Autoimmune disease**	yes		44	2.59 ± 1.88	0.0188	1.07 (0.83 – 1.39)	1.06 (0.83 – 1.36)
	no		519	2.25 ± 1.58	0.0166	1	1

Online Resource 2. The outcomes of negative binomial regression models used to explore the correlation between the presence of AID and the number of relapses and the use of disease-modifying

therapies (DMT) in RRMS patients. Those with autoimmune thyroiditis were excluded from the analyses. The results are presented as incidence rate ratios (IRR) with 95% confidence intervals (CI). [a] The model was adjusted for age at MS onset, sex, and the duration between the initial MS manifestation and the end of the follow-up period.
