# Supplementary material for: The impact of autoimmune comorbidities on multiple sclerosis progression: insights from a longitudinal single-center study
Source: J Neurol. 2025 Sep 3;272(9):607. doi: 10.1007/s00415-025-13351-2 (PMC12408772; doi:10.1007/s00415-025-13351-2)
Supplement: Supplementary file 3 — Supplementary file3 (PDF 124 KB) [file 415_2025_13351_MOESM3_ESM.pdf]

Journal: Journal of Neurology (Springer Nature)

The Impact of Autoimmune Comorbidities on Multiple Sclerosis Progression: Insights from a Longitudinal Single-Centre Study

Derya Aslan[a], Sabrina Bourabia[a], Bernd Kowall[b], Agne Straukiene[c], Konstantin Fritz Jendretzky[d], Franz Felix Konen[d], Thomas Skripuletz[d], Aksel Siva[e], Mehmet Fatih Yetkin[f], Tim Hagenacker[a], Christoph Kleinschnitz[a], Refik Pul[a,*], Jelena Skuljec[a]

[a] University Medicine Essen, Department of Neurology, Center for Translational Neuro- and Behavioral Sciences (C-TNBS), Essen, Germany.

[b] Medical Faculty, University Duisburg-Essen, Institute for Medical Informatics, Biometry and Epidemiology, Essen, Germany.

[c] Torbay and South Devon NHS Foundation Trust, Department of Neurology, Torquay, United Kingdom; University of Plymouth, Plymouth, United Kingdom.

[d] Hannover Medical School, Department of Neurology, Hannover, Germany.

[e] Istanbul University, Cerrahpaşa School of Medicine, Department of Neurology, Clinical Neuroimmunology Unit & MS Clinic, Istanbul, Turkey.

[f] Erciyes University, Faculty of Medicine, Department of Neurology, Kayseri, Turkey.

*Corresponding author: Prof. Refik Pul, MD; E-mail: refik.pul@uk-essen.de

		N	Annual total dosis of steroids	Average bootstrap estimate with percentile interval [a, b]
**Autoimmune disease**	yes	44	1.81 ± 2.33	-0.80 (-1.67, 0.02)
	no	520	2.66 ± 6.56	
		**N**	**Number of plasma exchanges**	**Average bootstrap estimate with percentile interval [a, b]**
**Autoimmune disease**	yes	44	0.68 ± 2.05	-0.02 (-0.56, 0.68)
	no	519	0.69 ± 2.11	
		**N**	**Delta EDSS**	**Average bootstrap estimate with percentile interval [a, b]**
**Autoimmune disease**	yes	40	0.24 ± 1.17	-0.12 (-0.52, 0.28)
	no	498	0.29 ± 1.35	

Online Resource 3. The outcomes of 1,000 bootstrapped linear regression models used to explore the correlation between AID and various outcomes in RRMS patients. Those with autoimmune thyroiditis were excluded from the analyses. Delta EDSS: difference between the last and the first recorded EDSS.

[a] The model was adjusted for age at MS onset, sex, and the duration between the first manifestation of MS and end of follow-up (in months); [b] The 2.5% and 97.5% percentiles were used for the analysis.
