# Supplementary material for: The impact of autoimmune comorbidities on multiple sclerosis progression: insights from a longitudinal single-center study
Source: J Neurol. 2025 Sep 3;272(9):607. doi: 10.1007/s00415-025-13351-2 (PMC12408772; doi:10.1007/s00415-025-13351-2)
Supplement: Supplementary file 4 — Supplementary file4 (DOCX 19 KB) [file 415_2025_13351_MOESM4_ESM.docx]

**Journal: Journal of Neurology (Springer Nature)**

**The Impact of Autoimmune Comorbidities on Multiple Sclerosis Progression: Insights from a Longitudinal Single-Centre Study**

Derya Aslan^a^, Sabrina Bourabia^a^, Bernd Kowall^b^, Agne Straukiene^c^, Konstantin Fritz Jendretzky^d^, Franz Felix Konen^d^, Thomas Skripuletz^d^, Aksel Siva^e^, Mehmet Fatih Yetkin^f^, Tim Hagenacker^a^, Christoph Kleinschnitz^a^, Refik Pul^a,^*, Jelena Skuljec^a^

^a^ University Medicine Essen, Department of Neurology, Center for Translational Neuro- and Behavioral Sciences (C-TNBS), Essen, Germany.

^b^ Medical Faculty, University Duisburg-Essen, Institute for Medical Informatics, Biometry and Epidemiology, Essen, Germany.

^c^ Torbay and South Devon NHS Foundation Trust, Department of Neurology, Torquay, United Kingdom; University of Plymouth, Plymouth, United Kingdom.

^d^ Hannover Medical School, Department of Neurology, Hannover, Germany.

^e^ Istanbul University, Cerrahpaşa School of Medicine, Department of Neurology, Clinical Neuroimmunology Unit & MS Clinic, Istanbul, Turkey.

^f^ Erciyes University, Faculty of Medicine, Department of Neurology, Kayseri, Turkey.

*Corresponding author: Prof. Refik Pul, MD; E-mail: [refik.pul@uk-essen.de](mailto:refik.pul@uk-essen.de)

| Ethnic Group | Country of Origin | n | % of total |
| --- | --- | --- | --- |
| European |  | **379** | **77.5** |
|  | Germany | 329 | 67.3 |
|  | Poland | 21 | 4.3 |
|  | Bosnia and Herzegovina | 4 | 0.8 |
|  | Italy | 4 | 0.8 |
|  | Serbia | 3 | 0.6 |
|  | Ukraine | 3 | 0.6 |
|  | Hungary | 2 | 0.4 |
|  | Kosovo | 2 | 0.4 |
|  | North Macedonia | 2 | 0.4 |
|  | Bulgaria | 2 | 0.4 |
|  | Romania | 2 | 0.4 |
|  | Greece | 2 | 0.4 |
|  | Lithuania | 1 | 0.2 |
|  | Portugal | 1 | 0.2 |
|  | Slovenia | 1 | 0.2 |
| Asian |  | **64** | **13.1** |
|  | Turkey | 41 | 8.4 |
|  | Kazakhstan | 6 | 1.2 |
|  | Lebanon | 4 | 0.8 |
|  | Iran | 4 | 0.8 |
|  | Syria | 2 | 0.4 |
|  | Iraq | 2 | 0.4 |
|  | India | 2 | 0.4 |
|  | Sri Lanka | 2 | 0.4 |
|  | UAE | 1 | 0.2 |
| African |  | **11** | **2.2** |
|  | Morocco | 7 | 1.4 |
|  | Ghana | 2 | 0.4 |
|  | Tunisia | 1 | 0.2 |
|  | Somalia | 1 | 0.2 |
| Others |  | **35** | **7.2** |
|  | Russia | 5 | 1.0 |
|  | Mixed | 30 | 6.1 |
| Total Subjects with RRMS |  | **489** | **100.0** |

**Online Resource 4.** Frequency of ethnic backgrounds and countries of origin in the relapsing-remitting multiple sclerosis (RRMS) cohort. Patients were categorized based on their ethnic background and the countries from which they originated. Individuals who were either born in a specific country or had both parents originating from that country were listed accordingly. If a patient's parents came from different countries, this was classified as "mixed." The countries of origin for these mixed backgrounds included Austria, Czech Republic, Spain, France, Belgium, Netherlands, Croatia, Latvia, Uzbekistan, Pakistan, Nigeria, Kenya, the USA, and Jamaica.
